# Effects of Steam and Water Blanching on Drying Characteristics, Water Distribution, Microstructure, and Bioactive Components of *Gastrodia Elata*

**DOI:** 10.3390/plants12061372

**Published:** 2023-03-20

**Authors:** Yong-Kang Xie, Xing-Yi Li, Chang Chen, Wei-Peng Zhang, Xian-Long Yu, Hong-Wei Xiao, Feng-Yin Lu

**Affiliations:** 1 Research Center for Agricultural and Sideline Products Processing, Henan Academy of Agricultural Sciences, Zhengzhou 450002, China; 2 Department of Food Science, Cornell University, 630 West North Street, Geneva, NY 14456, USA; 3 School of Artificial Intelligence, Beijing Technology and Business University, Beijing 100048, China; 4 Agricultural Equipment Intelligent Technology Research and Development Center, Shandong Academy of Agricultural Machinery Sciences, Jinan 250100, China; 5 College of Engineering, China Agricultural University, Beijing 100083, China

**Keywords:** *G. elata*, steaming and blanching, drying, water variation, microstructure, bioactive component

## Abstract

In the current work, the effects of steam and boiling water blanching on the drying characteristics, water distribution, microstructure, and contents of bioactive substances of *Gastrodia elata* (*G. elata*) were explored. Results showed that the degree of steaming and blanching was related to the core temperature of *G. elata*. The steaming and blanching pretreatment increased the drying time of the samples by more than 50%. The low-field nuclear magnetic resonance (LF-NMR) of treated samples showed that the relaxation time corresponded to water molecule states (bound, immobilized, and free) and *G. elata* became shorter, which indicated a reduction in free moisture and increased resistance of water diffusion in the solid structure during drying. Hydrolysis of polysaccharides and gelatinization of starch granules was observed in the microstructure of treated samples, which was consistent with changes in water status and drying rates. Steaming and blanching increased gastrodin and crude polysaccharide contents and decreased *p*-hydroxybenzyl alcohol content. These findings will contribute to a better understanding of the effect of steaming and blanching on the drying behavior and quality attributes of *G. elata*.

## 1. Introduction

*Gastrodia elata* Blume (*G. elata*) is a plant in the Orchidaceae family. Its Chinese name is Tian-ma, and its underground tuber has been used as a herb for treating headache, epilepsy, tetanus, vertigo, convulsion, and other nervous disorders for thousands of years in many Asian countries [1]. Modern medical research shows that *G. elata* contains phenols (such as gastrodin, *p*-hydroxybenzyl alcohol, etc.), sterols (*β*-sitosterol, etc.), organic acids (citric acid, succinic acid, etc.), polysaccharides, and other bioactive compounds [2]. Phenols, in particular, have anticonvulsant, analgesic, hypnotic, sedative, neuroprotective, and other effects [3]. According to Chinese pharmacopeia [4], gastrodin is widely considered the primary phytochemical compound responsible for the medicinal functions of *G. elata* tubers. In recent years, gastrodin and *p*-hydroxybenzyl alcohol have become the focus of active research aiming to evaluate the therapeutic value of *G. elata* due to their significant pharmacological activities [5]. Liu [6] suggested that *G. elata* polysaccharide is another essential bioactive substance. Since then, *G. elata* polysaccharides have received extensive attention worldwide, with other studies reporting various pharmacological activities, such as anticancer, antivirus, and neuroprotective effects [7]. Because of its many reported uses, *G. elata* is a promising candidate for multiple applications in the fields of drugs, food, and health products.

Traditionally, freshly harvested *G. elata* tubers are soaked in water and washed to remove the surface dirt, boiled in water or steamed, and then dried. Steaming or blanching is an essential operation in its processing and is vital for quality formation. In addition, steaming or blanching treatment increases the content of gastrodin and prevents the browning of *G. elata* during drying by inactivating the polyphenol oxidase enzymes, which often trigger enzymatic browning reactions [8]. Previous studies have shown that the content of gastrodin in steamed or blanched *G. elata* is significantly higher than that in fresh samples [9]. In the steaming or blanching process, the degree of steaming or blanching is the crucial factor affecting quality. If *G. elata* is not thoroughly steamed or blanched, the precursor substances of gastrodin (the main active component) cannot be adequately transformed, resulting in a low content of gastrodin [10]. On the other hand, if *G. elata* is over-steamed, the epidermis of the tuber is damaged and starch is liquefied, which result in bioactive substance loss, longer drying time, and lower processing efficiency. In addition, over-steamed or over-blanched *G. elata* products have a red color after drying, which seriously affects the appearance. Therefore, it is important to determine the optimal degree of steaming and blanching for *G. elata* for the best quality and processing efficiency.

Previous research on the steaming of *G. elata* has mainly focused on the optimization of the steaming process and the mechanisms underlying changes to the active components during the steaming process. Ning et al. [11] compared the active components of *G. elata* after steaming and blanching. They found that the contents of gastrodin, *p*-hydroxybenzyl alcohol, and parishin in *G. elata* material after blanching were lower those in the steamed samples. Qin et al. [12] studied the difference in quality among traditional blanched, steamed, and directly dried *G. elata*. The results showed that the contents of gastrodin, alcohol extract, and *G. elata* polysaccharide were the highest in the steamed samples, followed by the traditionally blanched samples, and lowest in the directly dried samples. These previous findings suggest that only steaming or blanching fresh *G. elata* can promote the formation of bioactive compounds, and the steaming method is more conducive in improving the content of active components. Studies have shown that steaming temperature, time, and pressure are important factors affecting the content of bioactive compounds in *G. elata* [13,14]. Meanwhile, steaming is a complex process that changes the chemical composition of *G. elata* tubers. Wang et al. [15] studied changes in seven bioactive compounds in *G. elata* during the steaming process. Their results showed that the content of each component tended to be stable after 60 min of steaming. During the steaming process, the parishin glycosides were incompletely hydrolyzed to produce gastrodin, citric acid, and other parishin glycosides. Different researchers have found different mechanisms underlying these transformations [16,17], which has made it difficult to guide industrial production.

The literature lacks a detailed study on the water migration within the *G. elata* during the steaming and blanching process and its effect on drying characteristics. Therefore, the study was undertaken to conduct quantitative research on the degree of steaming and blanching in *G. elata*, followed by an understanding of the influence of steaming and blanching on its drying characteristics. Further, the rule of water migration during *G. elata* steaming and blanching was examined; the effects of steaming and blanching on the microstructure and on the contents of gastrodin, *p*-hydroxybenzyl alcohol, and crude polysaccharide of *G. elata* were studied.

## 2. Results and Discussion

### 2.1. Degree of Steaming and Boiling Water Blanching in G. elata

#### 2.1.1. Gelatinization Temperature of *G. elata* Powder

**Figure 1:**
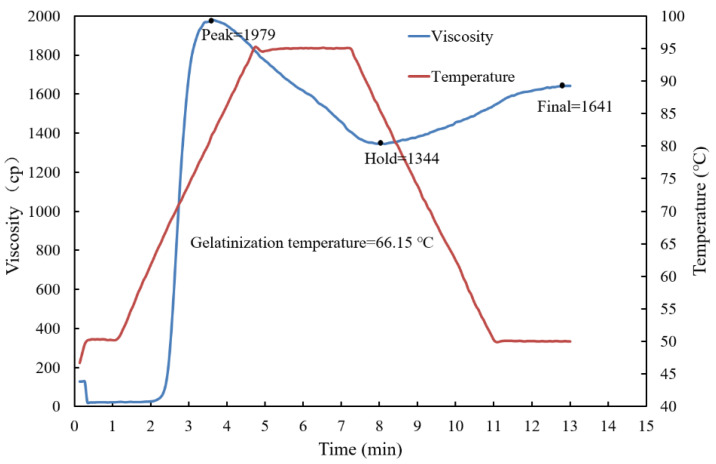
Viscosity characteristic curve of *G. elata* powder.

Gelatinization is one of the most important properties of starch, reflecting its capacity to absorb water and expand. In the processes of steaming and water blanching, gelatinization of starch is also affected by other components. Therefore, understanding the gelatinization characteristics of *G. elata* powder is of practical significance in exploring the degree of steaming and blanching of *G. elata*. The gelatinization temperature of *G. elata* powder was around 66.15 °C (viscosity value 73 cp), according to the viscosity (Figure 1), consistent with the gelatinization temperature of *G. elata* tissue solution (64–70 °C) found by Xie et al. [18]. The gelatinization temperature of *G. elata* powder was lower than the gelatinization temperatures of other agricultural products, which was possibly due to the particle size and morphology of the starch molecules. *G. elata* starch showed irregular polyhedron morphology and belonged to very small particle starch [18], which was consistent with a particle size distribution range of 615~1483 nm and the average particle size of 1185 nm found by Li et al. [19]. In addition, Goering et al. [20] found that small-granule starch generally had a lower gelatinization temperature than large-granule starch. Meanwhile, there were different opinions, which suggested that small starch granules were harder to break and the molecular arrangement order was more difficult to destroy, so they were not easy to gelatinize. This showed that the gelatinization temperature of starch might be the result of a combination of many factors, rather than simply depending on the size of starch granules. Furthermore, Li et al. [19] found that the amylose content of *G. elata* accounted for 7.25%, the amylopectin content accounts for 92.75%, and the branching degree was 3.72. The lower gelatinization temperature of *G. elata* starch might also depend on the content of amylose and the branching chain length of amylopectin. The long branching chain of amylopectin could also mimic amylose and stretch to support the integrity and stability of the whole particle structure, thereby inhibiting the gelatinization of starch [21]. Guan et al. [22] found that compared with non-steamed *G. elata*, the content of amylose in steamed *G. elata* increased by 1.08-times that of amylopectin, and the degree of molecular crosslinking was increased by steaming. These results might also indicate that *G. elata* amylopectin was starch with high branching degree but a short branching chain. Therefore, the low gelatinization temperature of *G. elata* was due to comprehensive factors.

#### 2.1.2. Relationship between Steaming and Blanching Degree and *G. elata* Central Temperature of the Cross Section with the Largest Diameter

**Figure 2:**
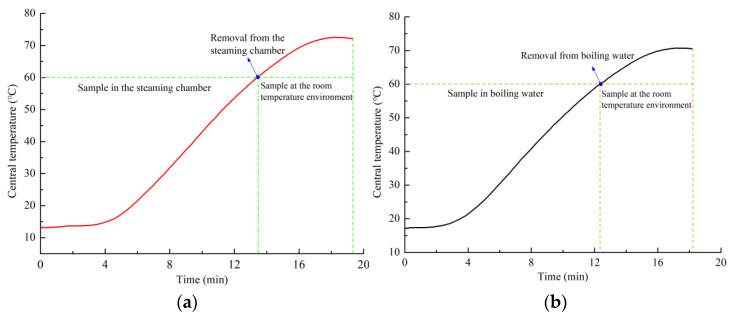
Temperature vs. time profiles of *G. elata* during and after steaming or boiling water blanching with a target temperature of 60 °C; (**a**) steaming; (**b**) boiling water blanching.

In the process of steaming or blanching, a proportion of white center in the section was used as an indicator of complete steaming or blanching. The steaming or blanching degree could be divided into three levels, namely, under-steamed, well-steamed and completely steamed. If the cross-section of *G. elata* had a white color area, it meant that it was under-steamed; if there was no white area or a little part of a white area in the cross-section, it meant that it had been steamed well; if there was no white area in the cross-section, it meant that it had been completely steamed. The ratio of white-color areas significantly decreased with increasing core temperatures, as shown in Table 1. When the real-time core temperature of *G. elata* was 45 °C, the white area in the center was 30.82% for steamed and 20.56% for blanched *G. elata*. When the real-time core temperature exceeded 60 °C, *G. elata* was thoroughly steamed or blanched, and the white area disappeared (the proportion of white area at the center was 0). A change in starch color mainly caused the color change in *G. elata* during steaming or water blanching. The temperature at which the white core area of *G. elata* disappeared was related to the gelatinization temperature of *G. elata* starch. When the real-time core temperature was 60 °C and the final core temperature was more than 66.15 °C (Figure 2), *G. elata* starch was gelatinized, and the section was completely free of white, indicating that *G. elata* was entirely steamed or blanched.

When *G. elata* was steamed until a target temperature of 60 °C was reached in the core region, the sample was immediately removed from the steaming chamber; afterwards, the core temperature continued to increase to 72.0 ± 1.0 °C (Figure 2a). The continuous temperature rise in the core region was known as thermal inertia, which was because the outer region of the tuber had higher temperatures when the sample was removed from the steaming chamber, from which the heat was transported to both the core region through conduction and the environment through convection. Similarly, after removal of the *G. elata* sample from the boiling water when the core region reached 60 °C, the internal temperature increased to 70.0 ± 1.5 °C (Figure 2b). Further, when the real-time target core temperatures of *G. elata* samples reached 45 °C, 75 °C, and 85 °C and then removed from the steaming chamber or boiling water, the final core temperatures were 59.5 ± 0.5 °C, 80.5 ± 1.5 °C, and 88.0 ± 0.5 °C, respectively, for steaming, and 61.0 ± 1.5 °C, 81.0 ± 1.0 °C, and 87.5 ± 1.0 °C, respectively, for boiling. The results showed that the longer the heating time (that is, higher core temperature), the smaller the difference between the core temperature of *G. elata* and the heating temperature, indicating that a thermal equilibrium was approached. At the same time, it could also be found that the steaming or blanched degree of *G. elata* in Table 1 has a great relationship with the central temperature. The steaming or blanched degree could be quantified by monitoring the material temperature.

### 2.2. Effects of Steaming and Boiling Water Blanching on Drying Characteristics

**Figure 3:**
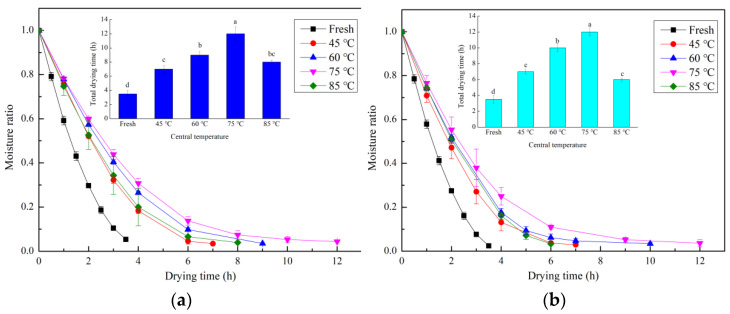
Moisture ratio curves of fresh, steamed, and blanched *G. elata* at different core temperatures of the cross-section with the largest diameter; (**a**) steaming; (**b**) boiling water blanching. Note: values followed by letters in the same figure indicate differences based on a Duncan test at a significance level of *p* < 0.05.

The drying time of fresh *G. elata* was significantly lower than that of *G. elata* samples after steaming and water blanching (Figure 3). As the core temperature increased, *G. elata* drying time increased and peaked at 75 °C then decreased with a further increase in core temperature. The drying times were 3.5 ± 0.5 h for fresh *G. elata* and 7.0 ± 0.5 h, 9.0 ± 0.5 h, 12.0 ± 0.5 h, and 8.0 ± 1.0 h, respectively, for samples steamed to core temperatures of 45 °C, 60 °C, 75 °C, and 85 °C. The drying time of *G. elata* samples steamed at 45 °C, 60 °C, 75 °C, and 85 °C increased by 50.0%, 157.1%, 242.9%, and 128.6%, respectively. In comparison, the drying times of hot-water-blanched samples to 45 °C, 60 °C, 75 °C, and 85 °C core temperature were 7.0 ± 1.0 h, 10.0 ± 0.5 h, 12.0 ± 1.0 h, and 6.0 ± 0.5 h, respectively. The drying time of *G. elata* samples steamed at 45 °C, 60 °C, 75 °C, and 85 °C increased by 50.0%, 185.7%, 242.9%, and 71.4%, respectively, relative to fresh *G. elata* samples. The drying mainly occurred in the falling rate stages, which suggested the dominating moisture transport mechanism during the hot air drying of *G. elata* was internal moisture diffusion. Fresh *G. elata* was dried significantly more rapidly than steamed or water-blanched *G. elata*. These results are consistent with the research of Xie et al. [18], who used high-temperature and high-humidity gas jet impingement steaming technology to treat *G. elata* and showed that, although steaming increased the material temperature, it also slowed the drying. *G. elata* has a high content of starch and other viscous substances. Steaming and boiling water blanching could destroy the grain structure of starch, promote the release of starch chains, and then enter the cytoplasm and tissue fluid. On the one hand, the molecular chain of amylopectin broke and generated short amylose. The short amylose pulled the broken amylopectin molecules and the original amylose molecules together through hydrogen bonds, forming a complex dense network structure (as shown in Figure 6C,M), which increased the molecular cross-linking degree and, thus, hindered the flow of water [22]. In addition, the high-viscosity starch also covered the surface of the dry material, which was additional resistance. On the other hand, in the gelatinization process of starch, the combination of water molecules and starch particles would also reduce the degree of freedom of water molecules, and it was not easy to remove water during the drying process [23]. When the central temperature was higher than 75 °C, the drying time of *G. elata* was shortened, which may be due to the excessive steaming or blanching of *G. elata*, resulting in cell damage, the loss of *G. elata* internal solutes, such as polysaccharide and starch, and the easier flow of water out of cells during drying.

### 2.3. Effects of Steaming and Blanching on Internal Water Distribution of G. elata

**Figure 4:**
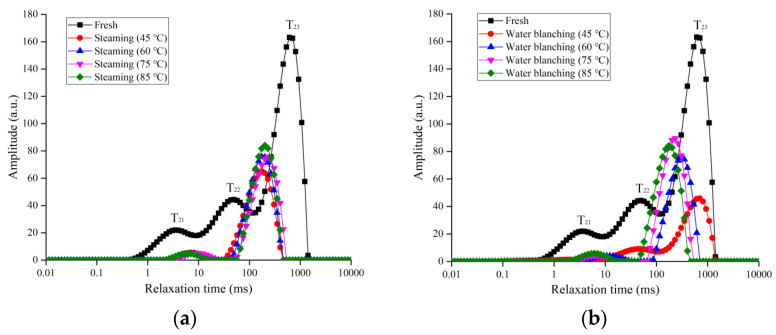
Transverse relaxation time curves of *G. elata* after steaming and water blanching at different central temperatures. (**a**) fresh and steaming; (**b**) fresh and boiling water blanching.

T_2_ relaxation time has often been used to study the distribution of and changes in water in cells. When the water molecules within the solid matrix have a higher degree of freedom, they usually have higher values of T_2_, corresponding to peaks on the right side of the T_2_ spectrum. On the other hand, when the water molecules have a lower degree of freedom, meaning that they had stronger binding force with the solid matrix, they have lower values of T_2_ and appear on the left side of the T_2_ spectrum. According to the different status of water molecules, T_21_, T_22_, and T_23_ represent the bound water, intracellular water, and intercellular water, respectively. Figure 4 shows the CPMG distribution of fresh, steamed, and boiled *G. elata* samples before drying. It was found that the free water molecules had the largest peak in the spectrum, suggesting that free water was the dominating status in *G. elata*. Since the free water had higher fluidity, it had the longest relaxation time. Hot water blanching and steaming led to decreases in peak height and a shift to lower relaxation times to the left side. Similarly, Chen et al. [24] found that after a block of *G. elata* was steamed, free water evaporated, and the relaxation time moved rapidly toward lower relaxation times.

Table 2 shows changes in relaxation time and relative areas of T_2_ peaks corresponding to fresh, steamed, and blanched samples. A_total_ is the water signal amplitude area of *G. elata*, representing the water content. The water content of *G. elata* decreased after steaming and blanching (Table 2), consistent with the finding by Xie et al. 2021 that the loss rate of *G. elata* increased after steaming. The relative areas A_21_, A_22_, and A_23_ represented the relative contents of bound water, intracellular water, and free water, respectively. A_21_, A_22_, and A_23_ changed significantly after steaming. A_22_ and A_23_, in total, contributed 90% of the total water content in the samples. Pretreatment led to increases in the relative area of A_23_ and decreased the relative areas of A_21_ and A_22_, which showed that the internal tissue structure of *G. elata* was destroyed after blanching and steaming, and the water diffused to the outside due to the internal and external pressure difference. The free water content also increased significantly. For *G. elata* materials, T_21_, T_22_, and T_23_ changed significantly, and the peaks moved to the left, indicating that the degrees of freedom of bound water, intracellular water, and intercellular water decreased after steaming and blanching, and it was difficult for water to exit the cells, consistent with the conclusion that blanching and steaming prolonged the drying time.

**Figure 5:**
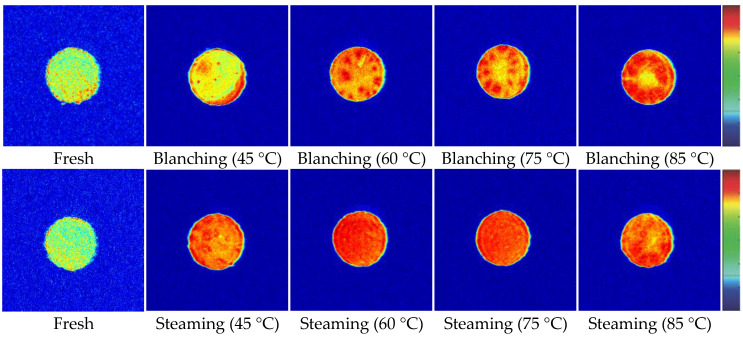
MRI image changes of *G. elata* during steaming and blanching.

A proton density diagram of the cross-section of the *G. elata* sample subjected to different steaming and blanching degrees is shown in Figure 5 with pseudo color. The color from blue to red represents the proton density in the sample from low to high. Fresh *G. elata* presents a high signal-to-noise ratio, and its water distribution could be clearly observed (Figure 5). The internal water distribution of fresh *G. elata* was uneven, and the water content gradually increased from the center to the edge. With an increasing core temperature of *G. elata*, the density signal of the central part of *G. elata* gradually increased, indicating that steaming and blanching led to the destruction of *G. elata* tissue structure. The edge water slowly diffused to the external environment, resulting in the uniformity of *G. elata* water. Steaming resulted in a more uniform distribution of water than blanching.

### 2.4. Effects of Steaming and Blanching on Microstructures

**Figure 6:**
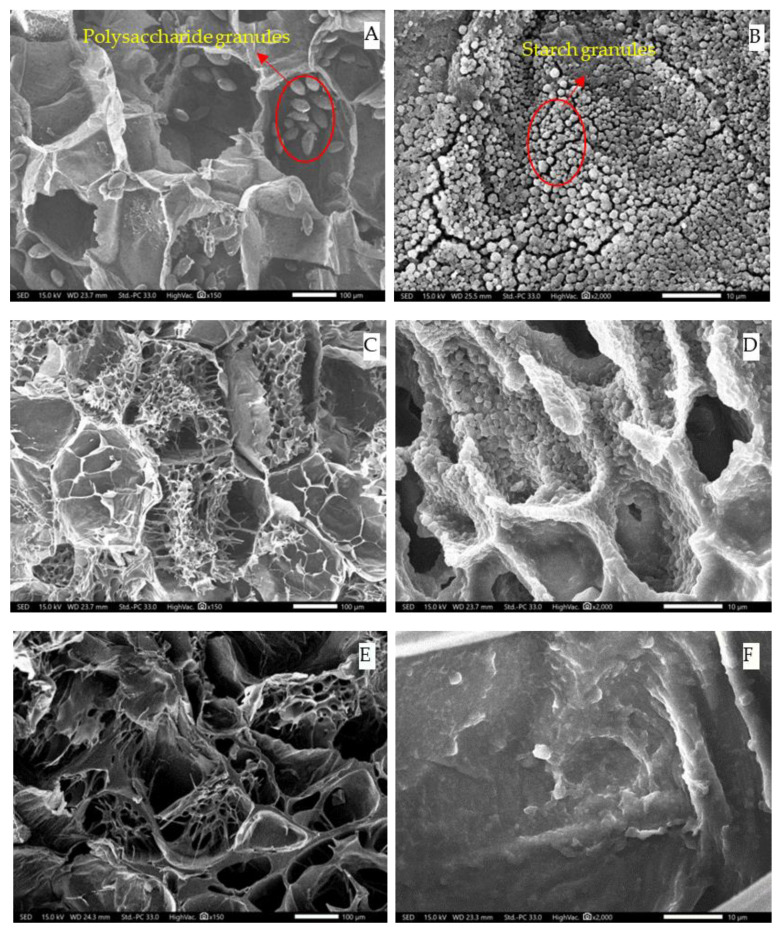

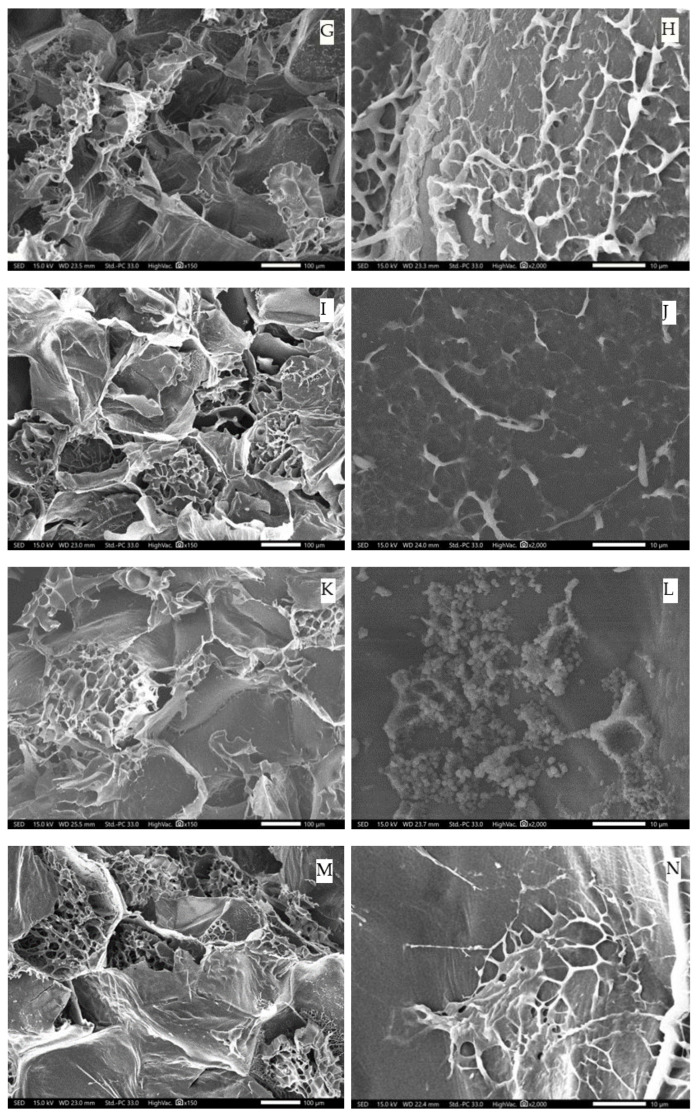

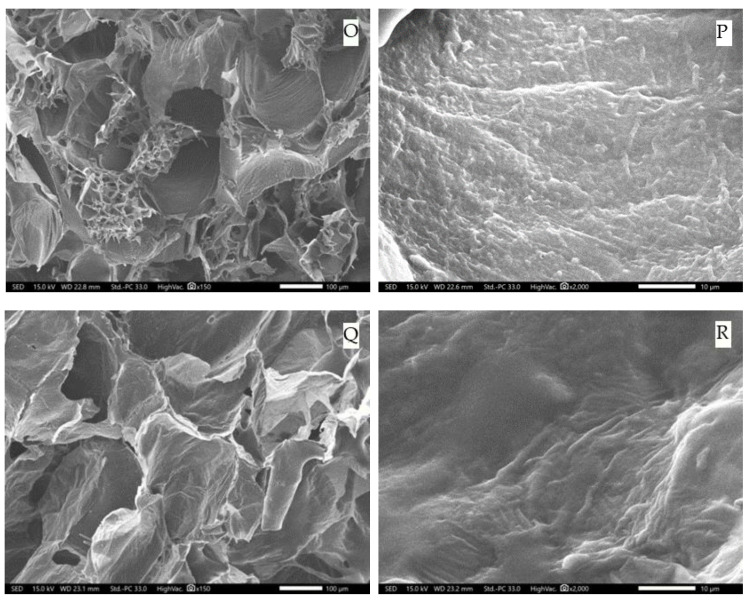
SEM micrographs of fresh, steamed, and water-blanched *G. elata* at different central temperatures of the cross-section with the largest diameter; (**A**,**B**): fresh *G. elata* at a magnification of 150× and 2000×, respectively; (**C**–**J**): *G. elata* that was steamed until a core temperature of 45 °C, 60 °C, 75 °C, and 85 °C at the cross-section with the largest diameter at a magnification of 150× and 2000×, respectively. (**K**–**R**): *G. elata* that was boiling water blanched until a core temperature of 45 °C, 60 °C, 75 °C, and 85 °C at the cross-section with the largest diameter at a magnification of 150× and 2000×, respectively.

The microstructure photographs of fresh, steamed, and blanched *G. elata* samples at different magnification (150× and 2000×) are shown in Figure 6. In fresh *G. elata* samples, the cells had an ordered and regular arrangement, and the cell membrane structure was complete. Furthermore, elliptical polysaccharide granules (150×) (Figure 3a) and irregular starch granules (2000×) (Figure 3b) were also observed in fresh *G. elata* cells. The diameters of *G. elata* starch granules were smaller, while the diameters of *G. elata* polysaccharide granules were larger. The starch and polysaccharide granules in this study were similar in shape and size to those observed in a previous study [18].

After steaming and blanching, the cell walls of *G. elata* were damaged, and some cells collapsed. The degradation of pectin in the middle layer may have led to the rupture of the cell walls, the softening of the tissue, and the loss of water in cells [25]. This result was consistent with the decrease in moisture content in the NMR signal amplitude after steaming and blanching treatment. In the low-magnification microstructure photographs (150×), it was observed that the elliptical polysaccharide disappeared with an increased core temperature of *G. elata*, resulting in the formation of intracellular mucus. However, the mucus disappeared when the core temperature of *G. elata* reached 80 °C. The cells were severely damaged, resulting in the loss of *G. elata* polysaccharide [26]. In high-magnification microstructure photographs (2000×), *G. elata* starch particles appeared to gradually gather together with increasing central temperature as *G. elata* starch began to gelatinize. When the core temperature exceeded the gelatinization temperature range of *G. elata* starch, the occurrence of *G. elata* starch particles decreased. When steamed or blanched to 85 °C, *G. elata* starch in cells disappeared. This was due to the liquefaction of *G. elata* starch after excessive steaming and blanching. In addition, during the process of steaming and water blanching, massive stickies adhered to cells, and it became difficult for water in the cells to migrate out of the *G. elata* tuber, resulting in an extension in drying time. When steaming and blanching were excessive, the lumps became less frequent or disappeared, and the water in the cells could easily migrate out, resulting in shortened drying time.

### 2.5. Effects of Steaming and Blanching on the Gastrodin, P-hydroxybenzyl Alcohol, and Crude Polysaccharide Content of G. elata

Compared to fresh *G. elata*, gastrodin and crude polysaccharide content increased after steaming and blanching (Table 3). In contrast, the content of *p*-hydroxybenzyl alcohol decreased, consistent with a previous study on *G. elata* steaming. Gastrodin content increased first and then decreased with increasing central temperature. When the core temperature of steamed and blanched *G. elata* reached 75 °C, the content of gastrodin was the highest, at 3.09 mg/g dry matter and 3.26 mg/g dry matter, respectively. The *p*-hydroxybenzyl alcohol content decreased with increasing core temperature. When the core temperature of steamed and blanched *G. elata* was 85 °C, the content of *p*-hydroxybenzyl alcohol was the lowest, at 0.44 mg/g and 0.53 mg/g, respectively. The maximum reduction in *p*-hydroxybenzyl alcohol was 58.14%. The crude polysaccharide content first increased and then decreased with increasing central temperature. With increasing core temperature, the content of crude polysaccharide reached its maximum when *G. elata* was blanched and steamed to a core temperature of 60 °C. During steaming and blanching, gastrodin was hydrolyzed to produce *p*-hydroxybenzyl alcohol and sugar through *β*-glycosidase, while *p*-hydroxybenzyl alcohol was condensed into gastrodin without enzyme participation. With increasing temperature at the center of *G. elata*, *β*-glycosidase enzyme activity gradually decreased, inhibiting the enzymatic hydrolysis reaction, but the condensation reaction was not affected. Therefore, the condensation of *p*-hydroxybenzyl alcohol to produce gastrodin resulted in increased gastrodin content and decreased *p*-hydroxybenzyl alcohol content. The increase in gastrodin was not equal to the decrease in *p*-hydroxyl, due to the degradation reaction of parishin during steaming and blanching, resulting in the increased content of gastrodin after steaming. It could be seen from Table 3 that the crude polysaccharide of *G. elata* increased after steaming or boiling water blanching. On the one hand, because the detection method is based on glucose as the standard, the starch, cellulose, and other substances in *G. elata* cells hydrolyze during steaming and boiling water blanching to produce reducing sugar, which increased the content after determination. On the other hand, with increasing central temperature, the cells were broken and the sugar flowed out of the cells, which improved the extraction rate of crude polysaccharides from *G. elata* [27].

## 3. Materials and Methods

### 3.1. Materials

The fresh, first-class *G. elata* root samples (weighing 200–250 g per individual, length was 94–113 mm, width was 50–62 mm, thickness was 37–52 mm) used in this study were collected at the plantation base of Bijie, Guizhou Province, China. All *G. elata* samples were stored at 4 ± 1 °C and 90% relative humidity before use. The sample’s moisture content before and after treatment was determined after drying in an oven at 104 °C until it reached a constant weight [4].

### 3.2. Steaming and Hot Water Blanching Treatment

Fresh *G. elata* samples were taken from the refrigerator and placed at room temperature for 6 h to allow them to reach room temperature. Before steaming or blanching, *G. elata* tubers were washed, and the surface water was removed using absorbent paper. Because of substantial variation in the sizes of *G. elata* of equal grade, the treatment time used to characterize the steaming or blanching degree had to be adjusted. Therefore, in the experiments, the core temperature was recorded at the cross-section of the maximum diameter of *G. elata* samples and used to characterize the degree of steaming. The temperature was measured using a T-type thermocouple (OMEGA Engineering Inc., Stamford, CT, USA) with an accuracy of 0.5 °C. The water in the steamer was heated by the electromagnetic furnace, and then the steam generated by boiling was used to steam the tubers of *G. elata*, so as to reach the predetermined target core temperature of 45, 60, 75, and 85 °C. For the hot water blanching experiments, the *G. elata* tubers were immersed in a thermostat water bath operated at 100 °C. After steaming and hot water blanching, all samples were cooled to room temperature quickly. The temperature sensor continuously monitored the core temperature of *G. elata*. All experiments were conducted under normal atmospheric pressure.

### 3.3. Hot Air Drying Experiments

The fresh, steamed, and blanched *G. elata* samples were cut into 5 mm-thick slices and transferred to the hot air dryer. The air temperature was 60 °C, and the air velocity was 0.5 m/s. Then, the effects of different degrees of steaming or blanching on drying were studied. Drying was stopped when the moisture content of *G. elata* was lower than the safe moisture content (12% wet basis). During drying, the drying data were recorded at predetermined time intervals, and moisture ratio (*MR*) was calculated. *MR* was calculated using Equation (1) [28,29]:

(1) $$MR=\frac{M_{t}}{M_{0}}$$

where *M*_t_ represents the moisture content at drying time *t* on a dry basis (kg·kg^−1^); *M*_0_ represents the initial moisture content on dry basis (kg·kg^−1^).

The drying rate (*DR*) was calculated according to Equation (2) [30,31]:

(2) $$DR=\frac{M_{t_{1}}-M_{t_{2}}}{t_{1}-t_{2}}$$

where *t*_1_ and *t*_2_ are the drying time (h); *M_t_*_1_ and *M_t_*_2_ are the moisture contents at time *t*_1_ and *t*_2_ on a dry basis, respectively (kg·kg^−1^).

### 3.4. Color Evaluation of Treated G. elata

At different core temperatures, the cross-sectional areas with white color in the core region of treated *G. elata* samples were assessed using a two-dimensional image based on the color difference. The white area of materials was analyzed and calculated by image segmentation and processing algorithm. The proportion of the white-colored area was calculated according to Equation (3):

L = S_1_/S_0_ (3)

where *L* represents the ratio of the white-colored area (%); *S*_0_ is the total cross-sectional area of *G. elata* (mm^2^); and *S*_1_ represents the white-colored area of the *G. elata* cross-section (mm^2^).

### 3.5. Determination of Gelatinization Temperature Sample Preparation

The *G. elata* roots were cut into 5 mm-thin slices and transferred to the pre-cooling chamber (−40 °C) by maintaining a medium freezing rate for 2 h. The frozen *G. elata* slices were quickly moved to the freeze dryer kept in a vacuum drying room (LGJ-10E, Ningbo Xinyi ultrasonic equipment Co., Ltd., Ningbo, China). The drying room was vacuumed after sealing, and the temperature control switch was turned on when the pressure was lower than 30 Pa. The shelf temperature during the freeze-drying process was 30 °C, and drying was stopped when the moisture content was lowered below 12% (wet basis). After freeze-drying, *G. elata* was crushed using a pulverizer (FW 135, Tianjin taist Instrument Co., Ltd., China) and screened using a 60-mesh screen.

The pasting property of *G. elata* whole powder (6% solids) was evaluated in triplicate using a Rapid Visco Analyzer (RVA-3D, Newport Scientific, Narrabeen, Australia). A programmed heating and cooling cycle was used, where the samples were held at 50 °C for 1 min, heated to 95 °C at a rate of 12 °C/min, maintained at 95 °C for 2.5 min, cooled to 50 °C at a rate of 12 °C/min, and then held at 50 °C for 2 min. Pasting temperature, peak viscosity, hot viscosity, final viscosity, breakdown viscosity (peak-hot viscosity), and setback viscosity (final-hot viscosity) were recorded [32].

### 3.6. Magnetic Resonance Measurements

LF-^1^H NMR measurements were performed using an NMR Analyzer (MesoMR23-060H-I, Niumag Corp., Shanghai, China) equipped with a 0.5 T permanent magnet corresponding to a proton resonance frequency of 20 MHz at 32 °C [33,34]. The *G. elata* tuber samples were placed in an NMR tube with an outer diameter of 40 mm. The proton decay signals were collected using the Carr–Purcell–Meiboom–Gill (CPMG) pulse sequence with a τ-value (time between 90° pulse and 180° pulse) of 200 µs and with 90° and 180° pulses of 7.5 and 15 µs, respectively. Parameters for NMR measurement were set as follows: echo time (TE), 0.35 ms; waiting time (TW), 2000 ms. Data from 18,000 echoes were acquired using 8 repeated scans [35].

Relaxation time analysis and distributed exponential curve fitting were performed using MultiExp Inv Analysis software (Niumag Corp., Shanghai, China). Multi-exponential fitting analysis was performed on the relaxation data using a modified inversion algorithm to obtain improved fitting. The relaxation time and its corresponding water population (area ratio) from this analysis were recorded.

MRI was performed using an NMR Analyzer (MesoMR23-060H-I, Niumag Corp., Shanghai, China) equipped with a 25 mm radio frequency coil at 32 °C [36]. The parameters for MRI measurement were set as follows: slice width, 3 mm; slice gap, 1 mm; TR (time repetition), 2000; TE (time echo), 25 ms; average, 4.

### 3.7. Microstructure Analysis

The microstructures of the fresh, steamed, and blanched samples were observed using a scanning electron microscopy (SEM, SU3500, Hitachi, Ltd., Tokyo, Japan). The pre-freezing and drying conditions of the sample were the same as those in Section 3.5. Samples were vacuum freeze-dried to a safe moisture content (12%) and cut into 4 mm cubes. Each sample was sputter-coated for 90 s with gold and analyzed using SEM at an accelerating voltage of 3.0 kV [37,38].

### 3.8. Measurement of Gastrodin Content and P-hydroxybenzyl Alcohol Content

The contents of gastrodin and *p*-hydroxybenzyl alcohol in *G. elata* were determined using high-performance liquid chromatography on an Ultimate 3000 standard liquid chromatography system (DIONEX, USA). A C18 column (4.6 × 250 mm, 5 m, Shimadzu, Japan) was employed in the analysis. The column temperature was 25 °C, and the injection volume was 20 mL. The mobile phases were 0.05% trifluoroacetic acid water (A) and 0.05% trifluoroacetic acid acetonitrile (B). The flow velocity was set at 0.8 mL/min, with the following elution gradients: 0–60 min, 95% A; 60–70 min, 70% A. Ultimate3000 Photodiode array detector was used at a detection wavelength of 220 nm. The results were expressed as mg/g dry matter. The detailed methods are reported by Gong [39].

### 3.9. Determination of Crude Polysaccharide Content of G. elata

Dried *G. elata* was crushed in a pulverizer for 2 min, and the *G. elata* powder was sieved through a 60-mesh screen. Then, 0.5 g of the sieved sample was weighed (accurate to 0.001 g), placed in a 50 mL plugged centrifuge tube, and 25 mL of deionized water (material liquid ratio 1:50 g/mL) was added. The mixture was shaken in a vortex oscillator to mix the contents fully. The sample was then extracted in an ultrasonic extractor (100 W) for 30 min with an initial ultrasonic temperature of 60 °C. After extraction, it was cooled to 25 °C, filtered, and the filtrate was transferred to a 100 mL volumetric flask. The residue was washed 2–3 times with deionized water, and the total volume was made up to 100 mL with water. This solution was the sample determination solution.

At the start, 0, 0.2, 0.4, 0.6, 0.8, and 1.0 mL of 100 mg/L standard glucose solution were added to 20 mL glass tubes with stoppers, and distilled water was added so that the final volume was 1 mL. Then, 1 mL 5% *v*/*v* phenol solution, followed by 5.0 mL of 98.08% sulfuric acid, was added, and the mixture was allowed to stand for 10 min. The solution was fully mixed using a vortex oscillator, then moved to a water bath at 30 °C for 20 min. The absorbance was then measured at 490 nm. The glucose mass was taken as the abscissa and the absorbance value as the ordinate, and a standard curve was drawn. The regression equation y = 0.0567x + 0.0339 with r^2^ = 0.9986 was obtained for drawing the standard curve.

The solution to be tested (0.5 mL) was sampled, and absorbance was detected according to the method in “drawing standard curve (y = 0.0567x + 0.0339)”. The glucose mass fraction value of the sample was calculated according to the standard curve equation.

### 3.10. Data Analysis-Statical Analysis

The experimental data’s mean and standard deviation (SD) were calculated from three independent replicates. Data were analyzed using ANOVA with post hoc Duncan’s multiple comparison tests using SPSS statistics 20.0 (International Business Machines Incorporation, USA) at the 95% confidence level.

## 4. Conclusions

The results of the present study showed that *G. elata* starch gelatinization could be confirmed from the internal color change. The relationship between the core temperature of the *G. elata* section and the proportion of white at the center showed that the real-time core temperature of *G. elata* without a white area was 60 °C. Steaming and blanching significantly decreased the drying rate of *G. elata*. After steaming and blanching, the degrees of freedom of binding water, intracellular water, and intercellular water of *G. elata* decreased, making it difficult for water to exit the cells. Further, cell wall collapse, starch gelatinization, polysaccharide denaturation, and hydrolysis could be observed in SEM. After steaming and blanching, the content of gastrodin and crude polysaccharide of *G. elata* increased, while the content of *p*-hydroxybenzyl alcohol decreased. When the core temperature was 75 °C, gastrodin content reached its maximum, and when the core temperature was 60 °C, the crude polysaccharide content of *G. elata* reached its maximum. Because the bioactive components were readily soluble in water, steaming was more suitable than blanching for processing, and the contents of the active ingredients were highest when the central temperature was 75 °C. These findings will contribute to a better understanding of the effect of steaming and blanching on the drying behavior and quality attributes of *G. elata*.

## Figures and Tables

**Table 1:** *G. elata*’s cross-sectional view and percentage of the white-color area at different central temperatures with the largest diameter of *G. elata*.

Central Temperature (°C)	Steaming			Boiling Water Blanching		
	Cross-Sectional View	Ratio of White Color Area (%)	Viscosity (cp)	Cross-Sectional View	Ratio of White Color Area (%)	Viscosity (cp)
Fresh	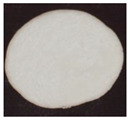	100.00 ± 0.00 ^a^	9.2 ± 3.5 ^e^	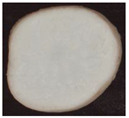	100.00 ± 0.00 ^a^	9.2 ± 3.5 ^e^
45	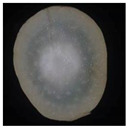	20.56 ± 3.43 ^b^	25.0 ± 1.0 ^d^	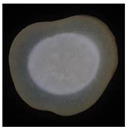	30.82 ± 1.97 ^b^	24.0 ± 1.0 ^d^
60	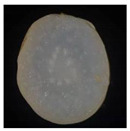	0 ^c^	1247.0 ± 4.0 ^c^	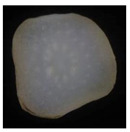	0 ^c^	1035.0 ± 15.0 ^c^
75	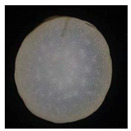	0 ^c^	1970.0 ± 2.5 ^a^	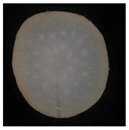	0 ^c^	1970.0 ± 1.5 ^a^
85	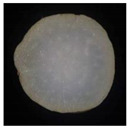	0 ^c^	1921.0 ± 3.0 ^b^	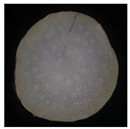	0 ^c^	1940.0 ± 5.5 ^b^

^a–e^: Different lowercase letters in the same column indicate significant differences (*p* < 0.05).

**Table 2:** Changes in relaxation time and peak area of *G. elata* before and after steaming and boiling water blanching.

Treatment	Central Temperature (°C)	A_Total_ (g)	A_21_ (%)	A_22_ (%)	A_23_ (%)	T_21_ (ms)	T_22_ (ms)	T_23_ (ms)
Fresh		2668.17 ± 33.50 ^a^	10.06 ± 0.09 ^a^	20.40 ± 0.02 ^a^	69.54 ± 0.00 ^d^	4.44 ± 0.30 ^a^	69.08 ± 0.00 ^a^	705.48 ± 0.00 ^a^
Water blanching	45	1075.61 ± 25.97 ^e^	3.61 ± 0.20 ^b^	20.67 ± 3.80 ^a^	75.51 ± 3.60 ^c^	2.01 ± 0.65 ^b^	43.29 ± 0.00 ^b^	705.48 ± 0.00 ^a^
	60	1269.74 ± 24.83 ^d^	0.60 ± 0.15 ^c^	5.54 ± 0.06 ^c^	93.86 ± 0.08 ^ab^	0.29 ± 0.04 ^c^	12.33 ± 1.5 ^c^	305.39 ± 0.00 ^b^
	75	1465.41 ± 19.05 ^b^	0.12 ± 0.04 ^d^	4.28 ± 0.04 ^d^	95.60 ± 0.01 ^a^	0.22 ± 0.00 ^c^	7.06 ± 0.00 ^d^	231.01 + ±0.00 ^c^
	85	1480.60 ± 29.33 ^b^	0.04 ± 0.01 ^e^	5.99 ± 0.02 ^c^	93.97 ± 0.04 ^ab^	0.19 ± 0.01 ^d^	6.14 ± 0.98 ^d^	174.75 ± 13.08 ^e^
Steaming	45	1258.16 ± 37.23 ^d^	0.03 ± 0.004 ^e^	6.99 ± 0.02 ^b^	92.99 ± 0.02 ^b^	0.19 ± 0.01 ^d^	7.06 ± 0.00 ^d^	174.75 ± 0.00 ^e^
	60	1364.00 ± 0.73 ^c^	0.01 ± 0.002 ^f^	5.69 ± 0.70 ^c^	94.30 ± 0.70 ^a^	0.19 ± 0.01 ^d^	7.06 ± 0.00 ^d^	174.75 ± 11.38 ^e^
	75	1373.69 ± 2.48 ^c^		4.84 ± 0.13 ^d^	95.17 ± 0.13 ^a^		7.06 ± 0.50 ^d^	200.92 ± 0.00 ^d^
	85	1479.61 ± 23.94 ^b^		4.99 ± 0.25 ^d^	95.01 ± 0.25 ^a^		7.06 ± 0.00 ^d^	200.92 ± 0.00 ^d^

^a–f^: Different lowercase letters in the same column indicate significant differences (*p* < 0.05).

**Table 3:** Gastrodin, *p*-hydroxybenzyl alcohol, and crude polysaccharide content of *G. elata*.

Treatment	Central Temperature (°C)	Content of Gastrodin (mg/g Dry Matter)	Content of *p*-Hydroxybenzyl Alcohol (mg/g Dry Matter)	Crude Polysaccharide (g/100 g Dry Matter)
Fresh		0.48 ± 0.01 ^d^	3.10 ± 0.16 ^a^	4.89 ± 0.54 ^d^
Boiling water blanching	45	1.63 ± 0.21 ^bc^	1.07 ± 0.17 ^b^	7.49 ± 0.52 ^ab^
	60	2.11 ± 0.38 ^b^	0.82 ± 0.17 ^bc^	8.38 ± 0.46 ^a^
	75	3.26 ± 0.03 ^a^	0.74 ± 0.09 ^bc^	7.56 ± 0.08 ^b^
	85	1.33 ± 0.16 ^c^	0.53 ± 0.06 ^c^	6.29 ± 0.21 ^c^
Steaming	45	0.73 ± 0.31 ^c^	1.18 ± 0.02 ^b^	8.04 ± 0.02 ^ab^
	60	1.49 ± 0.17 ^b^	1.04 ± 0.17 ^bc^	8.61 ± 0.01 ^a^
	75	3.09 ± 0.08 ^a^	0.69 ± 0.20 ^cd^	7.53 ± 0.13 ^b^
	85	1.22 ± 0.17 ^b^	0.44 ± 0.08 ^d^	6.26 ± 0.26 ^c^

^a–d^: Different lowercase letters in the same column of the same index indicate significant differences (*p* < 0.05).

## Data Availability

Data are contained within the article.
